# Enteric etiological surveillance in acute diarrhea stool of United States Military Personnel on deployment in Thailand, 2013–2017

**DOI:** 10.1186/s13099-020-00356-7

**Published:** 2020-04-09

**Authors:** Woradee Lurchachaiwong, Oralak Serichantalergs, Paphavee Lertsethtakarn, Nattaya Ruamsap, Apichai Srijan, Wirote Oransathid, Nuanpan Khemnu, Brian A. Vesely, Samandra T. Demons, Norman C. Waters, John M. Crawford, Brett E. Swierczewski

**Affiliations:** 1 Department of Bacterial and Parasitic Diseases, Armed Forces Research Institute of Medical Sciences, Bangkok, Thailand; 2 Bacterial Diseases Branch, Walter Reed Army Institute of Research, Silver Spring, MD USA

**Keywords:** Enteric etiology, Acute diarrhea, United States Military Personnel, Thailand

## Abstract

**Background:**

Diarrhea remains a major public health problem for both civilian and military populations. This study describes the prevalence of acute diarrheal illness etiological agents, their antibiotic resistance distribution patterns, the resulting impact upon military force health protection, and potential prevention and treatment strategies.

**Results:**

Forty-eight acute diarrhea stool samples from US military personnel deployed to Thailand from 2013–2017 were screened for enteric pathogens using ELISA, the TaqMan Array Card (TAC), and conventional microbiological methods. These isolates were also evaluated using antimicrobial susceptibility testing (AST) against ampicillin (AMP), azithromycin (AZM), ceftriaxone (CRO), ciprofloxacin (CIP), nalidixic acid (NA), erythromycin (ERY), and trimethoprim-sulfamethoxazole (SXT) using commercial methodology. Susceptibility results were interpreted following the CLSI and NARM guidelines. Questionnaire data obtained from 47/48 volunteers indicated that 89.4% (42/47) reported eating local food and the most common clinical symptoms were nausea and abdominal pain (51%; 24/47). Multiple bacterial species were identified from the 48 stool samples with diarrhea etiological agents being detected in 79% (38/48) of the samples distributed as follows: 43.8% (21/48) *Campylobacter jejuni* and *Campylobacter* species, 42% (20/48) diarrheagenic *Escherichia coli*, and 23% (11/48) *Salmonella*. Co-infections were detected in 46% (22/48) of the samples. All *C. jejuni* isolates were resistant to CIP and NA. One *C. jejuni* isolate exhibited resistance to both AZM and ERY. Lastly, an association between exposure to poultry and subsequent detection of the diarrhea-associated pathogens *E*. *coli* and *P. shigelloides* was significant (*p *< *0.05*).

**Conclusion:**

The detection of *Campylobacter* isolates with CIP, AZM and ERY resistance has critical force health protection and public health implications, as these data should guide effective Campylobacteriosis treatment options for deployed military members and travelers to Southeast Asia. Additional research efforts are recommended to determine the association of pathogen co-infections and/or other contributing factors towards diarrheal disease in military and traveler populations. Ongoing surveillance and AST profiling of potential disease-causing bacteria is required for effective disease prevention efforts and treatment strategies.

## Background

Exposure to enteric pathogens is one of the major causes of diarrheal infections in both traveler and military populations [1]. Previous studies have reported that military personnel acquired infectious diarrhea during military exercises [2]. The risk of diarrheal infection is regionally dependent, particularly for civilian travelers and military personnel in transition from industrialized countries into developing countries [3, 4]. Reported incident rates for bacterial diarrheal disease in military and travelers caused by enterotoxigenic *Escherichia coli* (ETEC), *Campylobacter*, and *Shigella* were between 38 and 45% in previous reports from various countries [5]. Typical treatment for traveler’s diarrhea includes the use antibiotics to include ciprofloxacin, azithromycin and rifaximin [6]. However, enteric pathogens and their associated antibiotic resistance patterns evolve over time and vary by region [7, 8]; therefore, access to up-to-date data on the global epidemiology of present diarrheal agents and their respective resistances are vital for diminishing the risk of diarrheal infection [6].

There are five Pacific region countries with which the US has a functional security alliance, including Thailand. The Armed Forces Research Institute for Medical Sciences (AFRIMS), based in Thailand, has coordinated studies of deployed US military to Thailand (i.e., the annual US–Thai “Cobra Gold” joint military forces exercise) for several years. Documented studies from previous exercises in Thailand demonstrated that US soldiers suffer consistent diarrhea attack rates during their first few weeks in country [9–11]. Despite modern preventive methods, diarrhea remains a primary concern for force health protection and therefore mission success for deployed military personnel in Thailand. Thus, the main objective of this study was to report the prevalence and clinical symptoms of diarrheal etiologic agents and bacterial pathogen antimicrobial susceptibility (AST) patterns affecting deployed US military personnel in Thailand for Cobra Gold exercises conducted in 2013 to 2017. This information will be useful in formulating more effective prevention and treatment strategies for these acute illnesses in deployed US forces.

## Methods

### Study design

A prospective acute diarrhea study was conducted in February of each calendar year, 2013–2017, at the following field sites: Lopburi, Phitsanulok, Chonburi (Samaesarn/Utapao), and Chanthaburi (Baan Chan Khem). Diarrheal cases were defined as three or more loose stool in the previous 24 h, starting no more than 72 h before presentation, with concurrent clinical symptoms such as nausea, vomiting, and abdominal or bowel pain. After obtaining informed consent, US military service members who presented with these criteria and symptoms self-reported on an administered questionnaire the following: stool frequency and description, poultry exposure, local food consumption, and any additional clinical symptoms. Stool grading (formed, soft, loose, or watery) was assessed by US military medical staff. The stool grading “loose” is described the stools that appear softer than normal whereas “watery” is specified to the stools appearance that no solid pieces and all liquid. The study was approved yearly by the Walter Reed Army Institute of Research institutional review board, Silver Spring, Maryland, USA.

### Laboratory methods

#### Microbiological methods

Approximately 4–10 grams of stool was collected from each subject. Stool samples were tested for the presence of enteric bacteria pathogens by inoculating onto the following media: MacConkey agar (MC), Hektoen (HE), thiosulfate citrate bile salt sucrose (TCBS), modified semi-solid Rappaport–Vassiliadis (MSRV), modified charcoal cefoperazone deoxycholate agar (mCCDA), buffered peptone water, alkali peptone water and Preston selective enrichment broth. Cellulose acetate membrane (Sartorius, Germany) was used to filter inoculated stool samples on Brucella agar plate (BAP) with sheep blood. Subsequently, identification of *Shigella, Salmonella, Vibrio*, *Aeromonas, Plesiomonas, Yersinia, Campylobacter* and *Escherichia coli* was performed as previously described [12].

#### Antimicrobial susceptibility testing

Isolated enteric pathogens, except *Campylobacter* and *Arcobacter*, were evaluated by AST following standard Kirby-Bauer method to the following antibiotics, ampicillin (AMP), azithromycin (AZM), ceftriaxone (CRO), ciprofloxacin (CIP), nalidixic acid (NA) and co-trimoxazole (SXT), using commercially prepared discs according to the manufacturer’s instructions (Becton, Dickinson and Company, USA). Susceptibility results were interpreted following the Clinical and Laboratory Standards Institute (CLSI) guidelines [13]. *Campylobacter* and *Arcobacter* isolates were evaluated using E-tests (Biomérieux, NC, USA). The minimal inhibitory concentration (MIC) was defined as the lowest concentration of an antimicrobial agent that completely inhibited visible growth and was read at the point where the elliptical zone of inhibition intersected the MIC scale on the strip. Due to the limitation of CLSI guidelines for *Campylobacter*, the National Antimicrobial Resistance Monitoring System (NARM) 2013 criteria for AZM, CIP, erythromycin (ERY) and NA were followed for these isolates [14].

#### Multiplex polymerase chain reaction (multiplex-PCR)

Isolated 5–10 lactose fermenting colonies were inoculated onto MacConkey agar (MC) and Hektoen (HE) media, cultured for 18–24 h at 37 ℃, and sub-cultured onto trypticase soy agar (TSA) with 5% sheep blood. Lactose-fermenting colonies were picked for nucleic acid by boiling extraction method. Identification of diarrheagenic *Escherichia coli* (Enteropathogenic *E.coli* (EPEC) [15], Enteroinvasive *E.coli* (EIEC) [16], Enteroaggregative *E.coli* (EAEC) [17, 18], Enterotoxigenic *E. coli* (ETEC) [19–21], and Enterohemorrhagic *E. coli* (EHEC) [15, 22] was performed using multiplex PCR.

#### Enzyme-linked immunosorbent assay (ELISA)

Qualitative Enzyme-linked immunosorbent assay (ELISA) kits (TechLab, Inc., USA) were utilized to detect *Giardia lamblia, Cryptosporidium,* and *Entamoeba histolytica* in fecal specimens. ELISA kits (Ridascreen® and R-Biopharm; Germany) were used for the detection of rotavirus, astrovirus, adenovirus, and *Campylobacter* according to the manufacturer’s instructions.

#### TaqMan^®^ array card

Total nucleic acid was extracted from frozen stool using the QiaAmp stool DNA kit (Qiagen, Valencia, California) and used in the Enteric Pathogen TaqMan^®^ Array Card (TAC) as previously described [23]. Briefly, 40 μL of extracted nucleic acid from each stool sample was mixed with 60 μL of Ag-Path-ID One-Step RT-PCR kit (Applied Biosystems, Foster City, CA) and this mixture was loaded onto the eight ports of the TAC, sealed, and loaded into the ViiATM7 instrument (Applied Biosystems). TAC can detect the following pathogens: bacteria: *Aeromonas*, *Bacteroides fragilis*, *Campylobacter* (*C. jejuni* and *C. coli*), *Clostridium difficile*, EAEC, EPEC, ETEC, *Helicobacter pylori*, *Salmonella*, *Shigella*/EIEC, STEC, and *Vibrio cholera*, fungi: *Encephalitozoon intestinalis* and *Enterocytozoon bieneusi*, nematodes: *Ancylostoma duodenale*, *Ascaris lumbricoides*, *Necator americanus*, *Strongyloides stercoralis*, and *Trichuris trichiura*, protozoan parasites: *Cryptosporidium*, *Cyclospora*, *Entameoba histolytica*, *Giardia* A/B, and *Isospora* and viruses: adenovirus, astrovirus, norovirus GI/GII, rotavirus, and sapovirus [23]. Analysis of raw data files were processed using ViiATM7 software version 1.2.2 (Applied Biosystems) as previously described [23]. A threshold cycle (Ct) greater than 35 was used as the analytical cutoff (lower limit of detection).

### Statistical methods

Statistical analysis was conducted using IBM SPSS Statistics version 24.0. Chi squared tests were sued to determine if the association between patient questionnaire data and subsequent pathogen identification was significant.

## Results

A total of 48 acute diarrhea cases were enrolled from 2013 to 2017 (47 completed questionnaire), with 9 cases in 2013, followed by 8 (2014), 18 (2015), 9 (2016) and 4 (2017) cases respectively. 38% (18/47) of the stool samples were described as “loose”, 21% (10/47) as “soft”, 23% (11/47) as “watery”, 6% (3/47) as “no record’ and the remaining as “formed”. 89.4% (42/47) of the subjects consumed locally prepared food. Primary self-reported complaints included: 72% (34/47) abdominal pain, 64% (30/47) nausea and 34% (16/47) vomiting. Bowel movement frequencies varied between 2 and 20 times in 48 h. Seven patients suffered from bloody diarrhea and three of the seven presenting with bloody diarrhea accompanied by nausea, vomiting, abdominal pain, and bowel movement pain. The clinical presenting data has been described on Table 1.

**Table 1:** Clinical symptom summary of US military personnel deployed in Thailand from 2013–2017 with diarrhea

Year	Total number	Bloody diarrhea (case)	Nausea (case)	Vomiting (case)	Abdominal pain (case)	Bowel movement range (times)
2013	9	0	7	6	6	2–10
2014	8	2	7	4	7	4–20
2015	18	2	10	3	12	3–20
2016	9	2	5	1	6	4–20
2017	4	1	1	2	3	7–20

Pathogen detection for stool samples collected from 2013 and 2017 was performed using ELISAs and TAC. Microbiological culture and multiplex PCR were added to the diagnostic panel for samples collected in 2015–2017. Of the 48 acute diarrhea stool samples, enteric pathogens were identified in 79.2% (38/48) of the samples while an etiologic agent was not detected in 20.8% (10/48) of the stool samples. The pathogenic profile of the 48 study samples from 2013 to 2017 is summarized in Table 2. Briefly, the most common detected pathogen was *Campylobacter* spp. (43.8%; 21/48), followed by the diarrheagenic *E. coli* (42%; 20/48) and *Salmonella* spp. (23%; 11/48). The most common *Campylobacter* species was *C. jejuni* at 25% (12/48), whereas *Campylobacter* spp. was identified in 18.8% (9/48) of the samples. Of the twenty diarrheagenic *E. coli* cases, 65% (13/20) were EPEC, followed by 15% (3/20) ETEC, 15% (3/20) EAEC, and 5% (1/20 EIEC). *Salmonella* spp. and norovirus were detected in 23% (11/48) and 15% (7/48) of the stool samples respectively (Table 2). Additionally, *Vibrio cholera* and *V. parahaemolyticus* were detected in US military personnel stationed at the Chonburi province, a coastal city, in 2016.

**Table 2:** Summary of identified pathogens present in stool samples collected during 2013 to 2017 from US military personnel presenting with diarrheal disease while deployed in Thailand

Year	Pathogen detection	Detection rate (percent
*Campylobacter*	*Campylobacter jejuni*	12/48 (25%)
	*Campylobacter* species	9/48 (18.8%)
	Total	21/48 (43.8%)
Diarrheagenic E. coli*	EPEC	13/48 (27%)
	ETEC	3/48 (6%)
	EAEC	3/48 (6%)
	EIEC	1/48 (2%)
	Total	20/48 (42%)
*Salmonella* spp.	*Salmonella* group B	5/48 (10%)
	*Salmonella* group c	4/48 (8%)
	*Salmonella* species	2/48 (4%)
	Total	11/48 (23%)
Norovirus	Norovirus GII	5/48 (10%)
	Norovirus GI	2/48 (4%)
	Total	7/48 (15%)
*Plesiomonas shigelloides*		6/48 (13%)
*Aeromonas* species		6/48 (13%)
*Vibrio* species		3/48 (6%)
Rotavirus		2/48 (4%)
*Helicobacter pylori*		2/48 (4%)
*Shigella* species		1/48 (2%)
*Arcobacter butzleri*		1/48 (2%)
No pathogen detected		10/48 (21%)

*Diarrheagenic *E.coli* [EPEC = Enteropathogenic *E. coli*, ETEC = Enterotoxigenic *E. coli*, EAEC = Enteroaggregative *E. coli*, EIEC = Enteroinvasive *E. coli*]

Co-infections (defined as more than one etiologic agent) were detected in 46% (22/48) of the study samples with one sample from 2016 containing seven enteric pathogens: *Aeromonas veronii* bv *sorbria*, *Arcobacter butzleri, C. jejuni*, EPEC, *Plesiomonas shigelloides*, *V. cholera,* and *V. parahaemolyticus*. Stool samples containing the diarrheagenic *E. coli* and *P. shigelloides* were found to be most commonly associated with those US service members who were exposed to poultry *(p *=* 0.02*). One surprising observation was the absence of any typical etiologic agents for 2/10 samples that were classified as bloody diarrhea.

All pathogenic bacterial isolates obtained from 2015 to 2017 were further sub-cultured to perform AST, with resulting antibiotic resistance profiles contained in Table 3. 100% of the *Salmonella* isolates were resistant to AMP and 44.4% resistant to SXT. 52.9% (9/17) of the diarrheagenic *E. coli* isolates were resistant to AMP and 100% (4/4) of the EPEC isolates resistant to SXT. One *C. jejuni* and one *A. butzleri* isolate were resistant to AZM. The AZM-resistant *C. jejuni* was also resistant to ERY. 100% of the *C. jejuni* isolates were resistant to CIP and NA. All of the *Plesiomonas* and *Aeromonas* isolates were susceptible to all tested antibiotics.

**Table 3:** Antibiotic susceptibility profile of enteric bacteria detected in stool samples from US military personnel presenting with diarrheal disease while deployed to Thailand from 2015–2017

Identified bacteria	Total isolates	Resistant isolates	Isolates with antibiotic resistance*						
			AMP	AZM	ERY	CIP	NA	SXT	CRO
*Campylobacter jejuni*	11	11	–	1	1	11	11	–	–
*Arcobacter butzleri*	1	1	–	1	0	0	0	–	–
*Salmonella species*	9	9	9	–	–	0	–	4	0
ETEC	3	2	2	–	–	0	–	0	0
EAEC	3	3	3	–	–	0	–	2	0
EPEC	11	4	4	–	–	1	–	4	0
*Shigella sonnei*	1	1	0	–	–	0	–	1	0
*Vibrio* species	3	2	1	–	–	0	–	0	–

– No antibiotic resistant detection

* *AMP* ampicillin, *AZM* azithromycin, *ERY* erythromycin, *CIP* ciprofloxacin, *NA* nalidixic acid, *SXT* trimethoprim/sulfamethoxazole, *CRO* ceftriaxone

## Discussion

Diarrhea remains a leading cause of acute morbidity and chronic health effects, negatively impacting the health and functionality of both traveler and military populations. US military service members often deploy into developing regions in which enteric pathogens associated with diarrheal disease are prevalent. *Campylobacter* was the most frequent pathogen identified in this study, which correlates to the high prevalence in travelers with acute diarrhea in previous studies in Thailand in travelers and US military service members participating in previous Cobra Gold exercises [5, 24, 25]. *Campylobacter* isolates from this study were also entirely resistant to quinolones (NA) and fluoroquinolone (CIP) antibiotics, which is of additional concern based upon recent evidence indicating that quinolone- and fluoroquinolone-resistant *Campylobacter* infections are associated with the development of post-infectious long term sequelae to include Guillen Barre Syndrome [26, 27]. *A.* butzleri, a member of the *Campylobacteraceae* family, was isolated from one stool samples. *Arcobacter* species are not typically associated with diarrheal disease, however, previous studies showed an 8% prevalence of traveler’s diarrhea associated with *A. butzleri* in Mexico, Guatemala, and India [28]. Study of tourist restaurants in Thailand suggested that *Arcobacter* was a food-borne pathogen and its isolates were frequently resistant to AZM which is the common therapeutic recommendation for the treatment of diarrhea in Asia [29]. The AZM-resistant *C. jejuni* was also resistant to ERY, the recommended antimicrobial treatment in invasive cases or to eliminate carrier states. Erythromycin resistance has been reported in Thailand previously [30].

The second most common etiologic diarrhea agent identified in this study was *E. coli*. ETEC is the leading cause of childhood diarrhea and the most frequent cause of diarrhea in travelers to developing countries [31]. ETEC contribution to diarrheal disease is dependent upon the region of interest and seasonality [32–34]. In this study, EPEC was detected more commonly in cases than ETEC. A previous study noted that EPEC was dependent upon co-infection with other pathogenic bacteria to include *Aeromonas* and *Salmonella* in travelers who developed travelers’ diarrhea [35]. However in our study, EPEC was detected in only one co-infected sample. Non-typhoidal *Salmonella* (NTS) was the third most common pathogen detected and previous epidemiological studies demonstrated that infection with drug-resistant NTS enterica serotypes was associated with excess morbidity [36]. Based on the antibiotic profiles in this study highlighted that AZM should remain first-line treatment for travelers’ diarrhea to Thailand [37]. Norovirus genogroups II and I were detected in several of the cases, but are usually associated with outbreaks of diarrhea. Nevertheless, previous studies have shown that norovirus is becoming commonly detected in both children and adults returning from tropical settings though most laboratories do not commonly test for norovirus in a hospital, clinical setting [38].

*Plesiomonas* and *Aeromonas* are not normally associated with travelers’ diarrhea though this study indicated that these pathogens were detected in samples with other enteric pathogens. These co-infection results, associated with clinical diarrhea in military patients, support evidence from previous studies that *Aeromonas* contribute towards the development of diarrhea [39]. Meng et al. reported that synergy or antagonism among pathogens likely affected the degree of diarrheal disease severity more than a single infection in children [40], and that the presence of multiple infections dramatically challenged the ability to properly identify the actual etiological agents of diarrhea disease.

There were several limitations to the study. A relatively small number of diarrheal stool samples were collected with no matched control sample which makes stating that the identified pathogen (s) were truly the cause of the diarrhea. Another limitation is the lack of antibiotic profiles for the bacterial pathogens detected in samples from 2013 to 2014 as the main diagnostic methodology used in these years were ELISAs and TAC. Inclusion of conventional microbiological methods allowed for the determination of antibiotic susceptibility profiles. Due to diagnostic limitations, some pathogens remain undetectable by these methods because they require challenging or unknown unfavorable growth conditions. A previous study indicates that *C. consisus* and *C. ureolyticus* are emergent-bacterial diarrheal pathogens [41]. However, these organisms are obligate anaerobes that require a H_2_-enriched atmosphere for optimum growth [42]. Methods to identify these pathogens were not used in this study.

## Conclusions

Ongoing diarrheal etiologic agent surveillance studies with antibiotic susceptibility testing should continue in large scale US military exercises these studies relay critical information necessary to protect traveler and military populations and to minimize diarrheal disease threats.

## Data Availability

Data sharing not applicable to this article.

## Completing interests

The authors declare that there is no completing interests.
